# Supplementary material for: Non-invasive smooth muscle electromyography (SMEMG) as a novel monitoring technology of the gastrointestinal tract of awake, free-moving pigs—A pilot study
Source: PLoS One. 2021 Sep 13;16(9):e0257311. doi: 10.1371/journal.pone.0257311 (PMC8437306; doi:10.1371/journal.pone.0257311)
Supplement: Attachment — Submitted filename: Response_to_Reviewers.docx [file pone.0257311.s002.docx]

**Response to Reviewers’ Comments and Suggestions**

**PONE-D-21-03411**

Non-invasive smooth muscle electromyography (SMEMG) as a novel monitoring technology of the gastrointestinal tract of awake, free-moving pigs – a pilot study

PLOS ONE

The authors would like to thank both the Editor and the Reviewers for considering the manuscript. All comments and recommendations have been thankfully accepted and corrections have been made accordingly. The changes are highlighted with Track Changes in the revised version of the manuscript. Please find our specific replies below.

**Reviewer #1:**

**The method used allowed for the assessment or measurement of the electrical activity of the muscles. However, she did not consider what the test result was about, what osbzar the obtained results were from, etc.**

Thank you for the comment of the Reviewer, however it is not clear for us what is the main point of this special note. The word “osbzar” is an obvious typo, but unfortunately, we cannot figure out the meaning of it. Regarding the consideration of the test results, we can say that the whole manuscript is about the explanation of the test results, so it is not clear for us what is the point here raised by the Reviewer. We kindly ask the Reviewer to give clear description about the concerns raised against our manuscript.

In the Introduction part of the manuscript, we described in detail the main objectives of the work and previous problems.

**There is no information of a slow wave or electrical discharges.**

The slow wave response was revealed on Figure 3, the whole methodological background is already published in our earlier manuscripts that are all cited in our recent publications (*Szűcs KF, Nagy A, Grosz G, Tiszai Z, Gáspár R. Correlation between slow-wave myoelectric signals and mechanical contractions in the gastrointestinal tract: Advanced electromyographic method in rats. J Pharmacol Toxicol Methods. 2016;82: 37–44. doi:10.1016/j.vascn.2016.07.005;* *Szűcs KF, Grosz G, Süle M, Sztojkov-Ivanov A, Ducza E, Márki A, et al. Detection of stress and the effects of central nervous system depressants by gastrointestinal smooth muscle electromyography in wakeful rats. Life Sci. 2018;205: 1–8. doi:10.1016/j.lfs.2018.05.015*). From these citations the Reviewer can get a wider insight to this methodology, but we cannot involve these earlier results into the current manuscript to avoid plagiarism. Since we filter out the fast electrical discharges during our record (previously we proved that these fast responses do not have significant impact on the mechanical contraction forces), therefore the slow electrical discharges are recorded as slow waves, only.

**It is not known at what stage of the MMC the analysis was performed and this is important.**

During our experiments we did 4-hour periods of measurements from 7 a.m. or 12.00 p.m. Since the animals have not been fasted, the stages of the MMC have no significance during the detection. Our aim was to measure the fed-induced alterations in the GI tract after meals. It is obvious that the MMC may had a contribution to the shape and frequency of slow waves, but their stages cannot be detectable clearly as it could be during/after fasting. Additionally, the 4-hour periods of detection very probably include the whole period of an MMC, thus we got a sum response instead of response of given stages. We think that our methods have higher practical importance providing a better insight to the effect of different fed on the GI tract motility in pigs.

**A number of biochemical and endocrinological factors play an important role in the regulation of electrical activity, there is no post-postal research in this field. No information is available on this.**

Thank you for this comment and we deeply agree that there are lots of factor that may modify the electric activity of the GI smooth muscle. However, such biochemical and endocrinological studies were not in the main scope of the study. Looking at Table 1 it becomes obvious that the main difference between the control and the test diet is the fiber content: the control diet did not contain any additional fiber sources while the test diet had added fiber content *(Opticell C5 concentrated fibre source: crude fibre: 660 g/kg DM, NDF: 854 g/kg DM, ADF: 725 g/kg DM, ADL: 82 g/kg DM, Agromed Austria GmbH, Kremsmünster, Austria)*. Since the participating barrow pigs were in a self-control study without significant endocrinological alterations, and the fiber itself has low impact on endocrinological and biochemical modifications (especially within a 5-day diet), therefore we do not think that the above mentioned factors should be measured in this special experiments.

**Reviewer #2**

**The authors presented an interesting article analyse myoelectric signals of the different GIT segments in growing and awake pigs and investigate the effect of fibre-rich nutrition on the GIT activity of pigs. Authors used non-invasive method for the measurement myoelectrical activity of stomach, small intestine and large intestine.**

Thank you very much for this comment.

**The authors report that “two diets (control and experimental) were administered in an alternating manner in four iterative cycles, each comprising an adaptation period (five days) and measuring day.” while in the table the results are presented as the absolute value for individual animals I think that authors estimated maximum of power spectrum density -minimum twice for each diet. I think that multiple measurements during the day (“measuring days” lasted for 4 hours per animal) make it possible to present the results as mean values for individual animals.**

Thank you for this comment and we would like to clarify this. In the Table 2 the presented results are mean values of the maximum of power spectrum density (PsDmax, mV2) changes for each individual animal (animal, 1, 2, …9). However, the Figure 4 shows the mean values of PsDmax of the control and experimental animals (n=9) examined in the test. The relevant section has been improved in the manuscript accordingly.

**The control diet (CTR) contained a low level of crude fibre (CF; 29 g/kg as in feed) while the experimental diet (EXP) had an increased CF content (49 g/kg as in feed). In table 1 there can be observed Fibre source - only for experimental diet on the level 38.60 and without fibre source for control diet. It seems that the sources of fiber in the control and experimental groups should be similar and differ only in the amount.**

The Table 1 shows that the main difference between the control and the experimental diet is the fiber content (29 g/kg as in feed *vs*. 49 g/kg as in feed, respectively): the control diet did not contain any additional fiber sources while the experimental diet had added fiber content (named *“Opticell C5” with crude fibre: 660 g/kg DM, NDF: 854 g/kg DM, ADF: 725 g/kg DM, ADL: 82 g/kg DM, producer: Agromed Austria GmbH, Kremsmünster, Austria*). We chose this kind of fiber source in our experiment because, (1) it is widely used in pig compound feeds in Europe, and (2) the crude fiber and fiber fractions (NDF, ADF) content of the product is constant and guaranteed. The used fiber source is also free of mycotoxins and other contaminants (e.g. heavy metals). At the beginning of the experiment, the most important nutrient content of the raw materials, including the fiber source, was evaluated in a proximate analysis. With the help of this concentrated fiber source, we had the opportunity to significantly increase the fiber content of the basic (control) compound feed without any differences in the main nutrients (such as protein, ether extract, minerals, and ileal digestible amino acids content) and energy content. The pigs in the experiment consumed the control and/or experimental diets without any residual feed (and every week the fed diets were adjusted to their body weight). Therefore, the differences (the absolute value of the maximum of power spectrum density) obtained in the experiment are solely due to the increased fiber (plus NDF and ADF-cellulose and hemicellulose) content of the test diet. The relevant section in the Materials and Methods has been extended with this explanation.

We thank once again the reviewers for their valuable comments that helped to improve the quality of our manuscript.
